# Abstract of the Proceedings of the Buffalo Medical Association

**Published:** 1867-05

**Authors:** T. M. Johnson

**Affiliations:** Secretary


BUFFALO

### Medical and Surgical Journal.

VOL. VI. MAY, 1867. No. 10.

### Original Communications.

ART. I—*Abstract of the Proceedings of the Buffalo Medical Association.*

TUESDAY EVENING, April 2d, 1867.

The Association was called to order by the President. Members present—Drs. Gould, Strong, White, Miner, Trowbridge, Lockwood, J. R. Lothrop, Shaw, Smith, Rochester, Gay, Jansen, Burger, F. W. Abbott, Brown and Johnson.

The minutes of the last meeting were read and approved.

By vote of the meeting the regular order of business was suspended, and the Treasurer's Report read.

On motion of Dr. White, the report was accepted and referred to the auditing board.

On motion of Dr. Samo, Dr. B. H. Dagget was invited to attend the meetings of the Association until the next meeting of Erie County Medical Society.

Dr. F. W. Abbott was elected to membership.

By vote of the Association the Treasurer was allowed to make a special report at some future meeting.

Disertations on designated subjects being in order, Dr. J. R. Lothrop read the following paper:

This paper will be devoted to an examination of some of the actions of mercury. I ought to say in the outset, that in this I do not aim to present anything of my own originating, aiming only to express as well as I may the opinions of others. These opinions will probably not be accepted by all, for they, in some measure, call in question beliefs long entertained and taught, and almost thought to be beyond question. It is always an ungracious task to combat what has been taken for granted, being supposed to rest upon well-founded facts, and to undertake to show that the facts are no facts. It is always unpleasant to attempt to unsettle beliefs, for many obvious reasons, but especially that it exposes to a charge of skepticism, in itself confessedly a state of mind not to be applauded or encouraged. But a glance at the history of medical and scientific inquiry will convince any one of this; facts have been taken for granted upon insufficient evidence, or being believed to be true, have so far controlled the mind as to have with it the force of truth itself, and thereby to make it incapable of detecting the error which underlies. In other words, error once taken for truth, has a most retarding influence upon all inquiry—for what is believed to be true dominates the mind as forcibly as what is absolutely true. I would not be understood to undervalue the only stable foundation in medicine, for the facts which we accept, viz: experience rightly interpreted. I only wish to keep before the mind the liability that exists to misinterpret experience, of which there is so much in medical inquiry, instructive to the earnest inquirer.

We all know how a preconceived idea warps inquiry—for under the leading of what we are prepared or wish to find true, even observation and experience, our only safe guides, mislead us; inclining us to feel, see, hear and believe not the actual, but the supposed—not the real error, but the seeming truth. I must allow, however, that when we trust ourselves to talk much about the "fallacies of experience," we are exposing ourselves to the hazard of unsettling beliefs which are well founded, and drifting into a wide sea of skepticism. There is no more just source of pride to scientific medicine, than the teaching of its accumulated experience. But at the same time, all will acknowledge that a reliance upon authority, in as far as it inclines the mind to rest, and represses independent thought, is to be deprecated.

With this extended introduction, which ought perhaps to be called a sort of begging of the question, I will enter upon the subject to be considered.

Mercury was known to the ancients and by them employed. Its use, however, was external only, in diseases of the skin and in parasitic affections. The older physicians used it, but with a salutary dread of the unpleasant results which followed its free employment. For the fact that it caused tremblings, paleness, wasting, and ulcers of the mouth, seems to have been known to Aristotle, and the Arabians in addition seemed to have been acquainted with its property of causing salivation. Europeans much later learned the use of it from the Moors of Spain and the Saracens during the Crusades, but then only externally or by fumigation. Later its powers in syphilis made it more generally known. Syphilis was attended with skin affections, and the fact probably led to a knowledge of its virtues as a special curative agent in this disease. This special curative action of mercury does not appear to me to be one of the fallacies of experience, but is entitled to have the sway of an actual fact. This I do not propose to question.

The internal use of mercury had its introduction if not its origin chiefly from Paracelsus and his followers, but still mainly confined to syphilis. Gradually its use was extended to other diseases. For we find it proposed and employed in inflammatory diseases, fevers, scrofula and induration, though there was not an agreement as to its benefits in all. By some it was extravagantly praised. Thus Belloste writing in 1695, speaks of it as "one of nature's miracles and a most rare gift of Providence." By others it was styled the Sampson of the Materia Medica. In modern times it has become a somewhat shorn Sampson.

Richter declared that the gradual extension of its use rested "far more upon a direct experience of its virtues than upon the scientific and often plausible theories invented to explain its operation, and which led to gross errors in practice." If this statement could be fully accepted and we could believe that its use had always been, or now always is, founded upon "direct experience of its virtues," the various uses of the remedy could not be called in question, and we should have less frequent occasion in witnessing its abuse, to regret that the earlier and simpler belief of its powers has not been maintained to the present. It is somewhat strange that upon the point of its absorption, a fact now so unquestioned, there should have been any difference of opinion. For upon this fact depend nearly all the actions which have been ascribed to it; at least all those actions which have been considered its special and most important ones. Yet Dr. Physick, in his time, wrote an essay to prove that it was not absorbed, but acted sympathetically. An instance of the misleading of theory, full of wholesome instruction.

Of a kindred nature was the theory that its salivary action must be carried to a great extent in order that its full benefits should be obtained. Boerhave caused his patients to spit three or four pounds in twenty-four hours, and Turner declared two or three quarts "a good and sufficient discharge;" and these undoubtedly were to them the lessons of experience. To this test also of experience could be referred that custom formerly so prevalent of subjecting all cases of fever to a salivation greater or less as essential, and an indication of recovery. The cases in which this action could not be induced were observed to be mostly fatal. The explanation, however, of this observed action, is now well enough known to be very different, and both the indication and practice to be without value.

Pereira classes mercury among spanæmics or medicines that promote secretion and exhalation generally, soften and loosen textures, check phlegmonous inflammation, lessen inflammatory effusions, and promote their re-absorption. As he expresses it more formally, "in my opinion mercury is an alterative and a liquefacient spanæmic." A good many of the above actions can be covered by the term alterative, and probably the definition is extensive enough to pretty well include modern ideas upon its action. We are well aware that the term alterative is made to cover a wide therapeutical ground, and one would have a long list, if he should write down the particular diseases in which the alterative action of mercury is sought for.

Whether mercury has all the properties above attributed to it, or not, I shall not now undertake to inquire. That some of the secretions are increased, in the face of the many cases in which an increased flow of saliva is perceptible to eyes even disposed to see what they look for, cannot be denied. As to its influence over secretion, it may also be safely said that its purgative action is in some way connected with this influence, either as cause or effect. It increases the secretion of the pancreas without doubt. It is probably true that it increases the secretion of the follicles of the intestines, and it may be, thus acts as a purgative; though most of its purgative influence is believed to depend upon its special action upon the liver, promoting a greater flow of bile, and thereby aiding or causing catharsis. Whether this belief is well founded is to some a matter of doubt, but of its purgative action, and through this, highly beneficial action, there can be no question. Whether it is specially beneficial as a purgative, or better than many, or any other purgative, depends very much on the belief of the practitioner who uses it. But while one may express a doubt whether its special benefits as a purgative, rest upon a secure foundation of observation, there can be no doubt that in many cases its purgative action is followed by most marked relief in certain cases.

In what has been said above, no definite distinction has been kept in view between secretion and excretion. The latter as well as the former we know, has many important and essential offices in the healthy working of the organs and functions of the body. Both, it may be admitted, are influenced if not specially, yet influenced by the action of mercury. Which effect is the most important it is not my purpose now to undertake to set forth. This is only alluded to in connection with the reputed action of mercury to cause absorption of fluids from the cavities. By whichever process, secretion or excretion, it is mostly effected, there have been few to doubt that the action is a real one and well established by facts.

It has been attributed to increased activity of the absorbents. On the other hand Headland thinks that mercury favors absorption and counteracts effusion, by its impoverishing effects upon the blood, thereby both weakening the force of the heart and diminishing the pressure on the blood-vessels. This is mainly an effect upon nutrition. But at present the fact is more important than the theory, and in view of all experience upon the subject, it may be questioned whether the fact exists. At least whether mercury as we would now a-days give it, is a helpful agent in the process. Of course effusions have various causes, as for instance mechanical, which unfit them to be acted upon by mercury, but the favorable cases do not exhibit always its expected benefits. The action of mercury in excess, as inhaling vapors habitually, causes wastings and disappearance of effusions, but the same is true of cholera, and what is a sufficient explanation of the latter, would not satisfy the accepted theory of action of the former. But as this opens too broad a field of inquiry, for the present time, I will limit myself to the examination of two, according to generally accepted doctrines, most prominent actions of mercury, viz: its cholagogue and its antiplastic properties.

The power of mercury to increase the secretion of bile has been thought to be too certain, to be questioned. The practice of a large number of physicians is based upon that supposed fact. Torpor of the liver, in certain conditions of illness, either as cause or consequence, or as a concomitant, has been assumed to exist, as a *definite functional derangement*, almost capable of demonstration. This taken for granted, a mercurial in larger or smaller doses has been deemed necessary, stimulating the faulty organ to its proper duty. The practice is based upon belief of the fact. It is not merely a belief that mercury increases all secretion, the bile among others, but that it acts in a special manner on the liver. Thus we find Dr. Wilson Philip writing: "Mercury has a special operation on the liver—a power not merely of exciting its functions, but of correcting the various derangements of that function, in a way which it does not possess with respect to any other organ, and which no other medicine possesses with respect to the liver." Perhaps there would be many who would not think that there were any facts to warrant so broad a statement; but yet, I think, it must be conceded that the number would be large to whom this special action of mercury in some dose would have the sway of an actual fact. As to the *method* by which this action is produced the agreement would be less general. Various theories of action have been proposed.

Some assume that it makes the bile more liquid, and thus promotes its flow; others that it directly stimulates the secreting cells of the liver; and still others that it liquifies the secretion of the bile duct, and thus favors the discharge. The second, viz: direct action on the liver itself, is the most commonly accepted method. But as before said, the belief accepted, upon what proofs does it rest? I suppose in the first place, most would say, experience proves it. Jaundice and enlargement of the liver, have more rapidly disappeared when it has been used, besides the discharges themselves have been evidence of this action. Bile has appeared abundantly in the fæcal evacuations after its use. This has been indicated by a change of color, in some cases a green, in others a dark color, being the indication of its presence. This is more especially convincing if just before, the stools were clayey, or, as commonly stated, free from bile. But this matter of color is less convincing, if the green color is due, as Dr. Thudicum and others assert, to an actual change in mercury itself, that is, becoming a sub-sulphide; or if the dark color is in all cases derived from the colon, as Dr. Inman believes. The latter from observation feels warranted in saying that the intestinal contents are never dark brown, or even deep yellow, but whitish, prior to their passage through the ileo-cœcal valve. In the colon, they get their brown fæcal hue. Clayey stools are not then a proof that the secretion of bile is arrested, but that the colon is functionally disturbed; and moreover, in deep jaundice, when no bile flows into the intestine, the stools are often dark. If dark stools succeed clayey, after a dose of mercurial, so they do after other purgatives, and even without purgatives; and if they prove anything, prove as well that mercury like other purgatives acts upon the colon, as that it acts upon the liver. Dr. Thudicum however, though he admits that cholochrome, or the coloring matter of bile, appears in healthy fæces, still denies that mercury increases the amount of bile, rather that it diminishes it. This opinion he puts forth based upon the experiments of Mosler, Scott, Nasse, Kölliker and Müller, who found that by calomel the bile was diminished. It is true that the experiments were made upon dogs, and the doses purgative. But inasmuch as other drugs have not acted differently on dogs and men, it may be inferred mercury would not; and though the doses were purgative, the faith in the cholagogue action of mercury, has not been limited to small doses, but has been equally great in all doses; so that if large doses are found to fail, small would be as likely to. It may be said that mercury may not increase the secretion of bile in a healthy, but it will do so in a diseased state of the liver. This argument will not apply to other organs. Diseased kidneys are not acted upon by medicines which are inoperative in health. Some such idea has been held, for Chapman says that free use of mercury may derange the liver—meaning I suppose a healthy one — and *produce* icterose affections, and Cheyne says that mercurials produce jaundice. Thus the agent which cures jaundice and enlargement of the liver, may cause them; not upon any homœopathic theory, but upon the theory of over-stimulation. Due allowance must be made, when mercury fails in jaundice, for the cases which arise from obstruction of the ducts and not from any fault in the secreting power of the liver, and thus not expect of it what it cannot reasonably be expected to accomplish.

After what has been said upon this branch of the subject, I think the action of mercury to increase the secretion of bile may be called in question. In reply to the statement that very decided relief does follow mercury, in disorders of the liver, either without or with general disorder, I might say that it could as well be got by any other purgative as active; and therefore at less cost. But the belief that mercury acts upon the liver is not confined to large doses. Small doses, frequently repeated, are believed to have the same action. This of course is founded upon experience and facts, as to jaundice, enlargements, and color of stools; like those which bear upon large, which have been noticed.

The second question in relation to mercury is, does it possess such antiplastic power as to give it a control over inflammation; i.e. to limit or remove its products? If we read most works on practice, and works which treat of inflammation of special organs, we shall find mercury about the first remedy proposed. In inflammation of the brain it has been deemed essential; likewise in acute inflammation of the lungs, heart, bowels, and by many, of the liver in some stages. In inflammation of serous membranes it is used to prevent adhesion, or effusion; in mucous membranes to prevent or soften exudations. Upon what theoretical grounds has this been accepted? I will pass over the various hypotheses of its action and state what is observed. When mercury has been for a long time taken internally, or when men or animals have been much exposed to its influence, certain conditions are observed to arise, which seem to depend upon some change in the blood. The tissues become soft, new formations disappear, healed wounds open. There is paleness, wasting and œdema; all, signs of impoverished blood. In other words, the blood is supposed to have lost a portion of its solid constituents, so that the amount is less than normal. It has therefore an unwonted fluidity and tends to escape; bleeding being common and sometimes excessive. These changes indicate a diminution of the material which is in excess in inflammation, and which furnishes the exudations observed as a result of it. It seems then pretty clear that, if mercury has the power to diminish the amount of the solid matter of the blood, *when they are normal*, it will equally diminish them when the amount is in excess, and thus prevent the admitted physical change produced by inflammation as a result of this excess of fibrin, viz: exudation upon membranes, or into the tissues of the solid organs. On the other hand, it equally by this very aplastic power, aids in the removal of those products of inflammation when once they are formed. For if by continued use it impairs the coagulability of the blood, and causes wasting of the solids of the *normal body*, it certainly seems highly probable that the *feebly* organized exudations of inflammation will give way before it. This argument is equally applicable to indurations and adventitious growths, such as tumors; being less highly organized than the normal structures, they must yield to a power that can act upon the higher formations. Such theoretical reasoning is more plausible than sound. For on the other hand it has been asserted, and it is without doubt true, that mercury has itself given rise to *plastic formations.* Equally plausible explanations on theoretical grounds can be given to account for it. Plastic formations are caused by fibrinous exudation, but for this last, it is not necessary that there should be an absolute excess of fibrin in the blood; proportionate excess is equally productive. This proportionate excess occurs when from any circumstance the number of red globules is diminished. The paleness observed in those who have taken mercury for any time, has certainly a very close resemblance to the pallor of anæmia, and hence seems to warrant the inference that in them there is a diminution of the number of red globules of the blood, and a relative increase of fibrin. The buffy coat which is so much relied upon as proof of the inflammatory state of the blood, appears equally in the blood of anæmics, and of mercurial anæmics. Hence, as in absolute excess of fibrin, there is ample reason to look for plastic formations. If we pursue the theoretical statement to its legitimate conclusion, there are equally good theoretical reasons for believing that mercury may *remove* fibrinous exudations; and *cause* then. Moreover some slight inference in the same direction, may be drawn from the fact that mercury is a cause of a disturbance of the system, if not inflammatory, at least of a febrile character, which is ordinarily supposed to add inflammatory elements to the blood. Thus, in whatever way we approach the subject *theoretically*, we find that the aplastic action of mercury is hard to establish by any rational explanation. If, however, we cannot tell the *how*, I presume there are enough who will not disbelieve the fact—or I should say the assumed fact—for nothing is a fact till it is indubitable.

But wherever *theory* may lead us, and whatever difficulties it may throw over the matter, *experience* is confidently appealed to. In the settlement of a therapeutic question, we can very seldom arrive at a certainty, i. e. we can not demonstrate it as we can a geometrical problem. The beliefs of careful and candid observers must then be allowed to influence the question, for those beliefs have the best foundation *possible* in the case, viz: experience. Dr. Latham probably expresses the convictions entertained by a great many physicians, accounted men of correct judgment, when he says, speaking of the treatment of acute affections of the brain, lungs, pleura, peritoneum, etc., "mercury does not supercede blood-letting; but aids its antiphlogistic powers, and yet spares its amount." The meaning of which is, that mercury aids bleeding or may serve as a substitute for it, in cases where it would not be deemed advisable. We might say that experience has convineed men that mercury is in its action more *destructive* than *constructive*, and when its destructive action is sought, it is in cases where a morbid product is to be got rid of.

In most instances, the argument from experience has been drawn from results not perceptible to the eye. In most cases men have not seen the process of removal. Acute diseases which end often in effusions, adhesions, contractions, and indurations, interfering with the proper movements and functions of organs, have, when treated by mercury, less frequently terminated with such results. Therefore it was maintained that the exudation at the bottom of such results, was prevented, or removed by the aplastic power of mercury. But in the single instance of iritis, the course of things was plain to the eye. In that disease the disappearance of plastic material poured into and upon the delicate structure of the iris could be watched, and the accelerating influence of mercury on the process, was supposed to be clearly visible. Men saw in this more even than the *hastening of removal;* they saw also the *limiting* power of mercury. The deposit was made less by it. Hence it was essential, and without it the worst results would fellow. Therefore no man dared leave mercury out of the treatment of iritis; and because the exudation was retarded or removed, and the worst results prevented, no question was raised as to the action. But Dr. Williams had the courage to treat a number of cases without mercury, and found that the results were about the same as to the amount of exudation, time of disappearance, and permanent effect upon the iris.

These cases are important, inasmuch as they call in question experience, and show how much it has been looking for results, rather than waiting for them. In other words, seeing what it was supposed would happen, rather than what actually did take place. And yet experience must not be thought valueless, with all its fallacies, for after all it is the best ground we have. If experience cannot settle these questions they will remain unsettled. How much it has to do, may be inferred from the fact that it has been thought to have established the truth of much that is now known to be error. The homœopathist appeals as confidently to experience as any one. He says, you may show the homœopathic theory to be groundless, and the infinitesimal theory absurd, but in practice the system is a success, i. e. experience shows it. Experience rightly interpreted shows no such thing. How, rightly to read experience is indeed a great matter, requiring the finest powers of mind, and an immense number of instances. But with all its difficulties it is the best means we have, and we must give the beliefs of the best minds, drawn from experience, the greatest weight in the decision of many questions.

I have in this paper meant to be understood, to entertain doubt about the cholagogue action of mercury as a special action. I

have meant also to express my almost total unbelief in its possession of aplastic powers, and my equal unbelief in its *usefulness*, from any such property, if it has it, in inflammation. I acknowledge its advantages as a purgative. I admit its power over secretion, especially the salivary. It acts powerfully, apparently on the glandular system. It cures syphilis, I am fully persuaded. I know of no theory of its actions which will explain them. I would not deride experience, only protest against hasty conclusions and wrong interpretations of it. No more harm can come from skepticism, than from such extravagant statements of its power as this, by an English physician, Dr. Martin, editor of Dr. Johnson's work on tropical climates. Speaking of its action in diseases of the liver, he says: "It is in fact by this very double action of purging and increasing the secretion at the same time, that calomel relieves the loaded and inactive vessels of the diseased gland, not to speak of the other acknowledged physiological influences of the mineral, such as its increase of all the secretions and excretions of the body; its influence on the capillary circulation; its febrifuge effect; the peculiar specific power attributed to it by physicians and surgeons as an antagonist to inflammations, whether general or local; its stimulant power over the absorbent functions; its power of unloading at the same time that it gives a new impulse to the vascular system; its *peculiar* power in removing viscid and tenacious intestinal secretions; its antiphlogistic, solvent, and alterative effects on the blood;—these are the actions and uses ascribed to mercury by the ablest British practitioners and authors, and they are such as to place this remedy second only in importance to blood-letting. I think the ablest American would hardly go as far as the ablest British practitioners are thus said to go. French and German practitioners are rather inclined to skepticism on the subject, and think the English and American physicians given over to extreme confidence. Yet Trousseau rebukes their incredulity, and says, there must be good ground for the confidence felt in the antiphlogistic power of mercury, and laments the prejudice of his countrymen against this "heroic remedy." That I run counter to the belief of many in what I have said, I am fully aware.

But as to the value of mercury in many important acute inflammaations, there is a difference among the best authors. To instance a few: In pericarditis, when acute, Graves says, and Stokes agrees, our best efforts will be unavailing, "unless they be succeeded by a speedy mercurialization of the system." But Dr. Markham says, "The actual influence which the remedy possesses over the disease has yet to be shown." Dr. Flint says that, experience has prepared him to take a decided position in opposition to the importance of this measure. Fuller has seen pericarditis come on during salivation, and therefore is not a believer in its power to cure. The real value of mercury in endo-carditis has yet to be shown, though many think it safer to use than to withhold it. Dr. Walshe considers mercuralization in acute pleurisy not inferior to bleeding, but many good physicians cannot see the need or advantage of either. While Gooch, Velpeau, Churchill, and others think mercury necessary in peritonitis, Dr. Meigs rejects it, trusting to bleeding; and Canstatt thinks mercury as useless as bleeding. I think we could find practitioners here who would agree with the last. In pneumonia while English physicians generally, think mercury essential, Grisolle says he would not venture to employ it; and Flint has never had reason to be dissatisfied with its disuse. I might continue citations from other sources, but as I have already occupied too much time and tried your patience, I will bring the paper to a close with the remark, that I am as anxious as any one to learn what experience does really teach, and to follow the leading of truth, ascertained truth, let it lead where it will.

At the conclusion of the reading of the paper, Dr. Gay moved that the thanks of the Association be tendered to Dr. Lothrop for his able and interesting paper. The vote was unanimous in the affirmative.

Dr. Rochester remarked that he had listened with interest to the paper just read, and did not arise to make any point or enter into an extended discussion of the subject, but must differ with Dr. L. in regard to one or two points. I think we should not be guided in regard to the action of mercury upon the human system by its action upon the systems of lower animals. Their organizations are so different and varied that the results are unreliable and may lead us astray. I cannot agree with the statement that the fecal matter in the small intestines is not changed in color by the use of mercury. I have seen fecal matter in the small intestine that was not of a light color. I have found the same matter in the gall bladder and in the small intestine. Dr. Williams is an enthusiast and his statements in regard to the treatment of iritis are not altogether reliable. Have myself tried to treat iritis without mercury, but have never succeeded as well as with it. Thorough examination and criticism is just and proper, but we must not go too far. Am much pleased with the paper and the fair manner in which the subject has been treated.

Dr. Strong said he was much interested in the paper which Dr. Lothrop had read before us. It seemed to be a very candid and philosophical resume of the historical state of mercury. Not to dwell upon the theories of its mode of operation on the specific organs and functions on which its powers are exerted, the history of the therapeutical use of mercury to my mind furnishes an eminent illustration of the evil consequences of *hobby-riding*. I suppose there will be but little dissent to this proposition, as it bears upon the old time heresies when nothing valuable was recognized in its effects in alleviating disease unless and until a free salivation was induced. Salivation being the measure and test of its value and the object aimed at. I suppose nearly all will admit now that the hobby-riding in this direction, to this extent, was rather a reckless feat. But as in everything else human, so in medicine—one extreme follows another. In this case, however, with the modification (as frequently happens,) that the extreme not only follows, but is distinctly begotten by the other. A medicine of such marked and peculiar potency could not be too freely and on too slight occasions used without producing undesirable if not pernicious results. These results would naturally excite prejudice, and prejudice naturally impairs vision, and with impaired mental vision we fail to see not only no good in its excessive and indiscreet use, but gradually come to distrust it and then to discard it altogether. These results have been reached by some authors and many practitioners. So that from being prescribed oftener perhaps than any one article of the materia medica, it has become not uncommon to read and to hear of its powers to control and obviate disease as being questionable or wholly ignored. *In medias res* seems to be especially appropriate in reference to the powers of mercury.

Instead of salivation furnishing any test of its virtues and to be desired, intelligent observations make it almost certain that all of its good qualities and its efficacy in treating disease may be secured without the least necessity of causing salivation. As to the testimony of authors in regard to it as quoted by Dr. L., I suppose few American practitioners are in the habit of deferring very much to the French in questions pertaining to our practical reasons in the treatment of disease. Unsurpassed, perhaps unequaled in physiological and pathological research, they seem to fail in the practical talent for combating disease by medication. As to another esteemed authority referred to as against the use of this article, I have sometimes feared that notwithstanding his general freedom from prejudice, or anything uncandid, (and in this respect I regard him generally as a model,) he has allowed his horror or disgust at the shocking abuse of mercurial preparations in certain cases to carry him to the other extreme of ignoring its positive merits in the alleviation and control of certain forms and phases of morbid processes. Now I think that some distinction needs to be made in the discussion of the merits of mercury. In a few diseases it may be said to be essential to their successful treatment. In a larger number while it may not be absolutely essential to final recovery; that is to say, patients may recover without it, yet mercury in some form is by far the most eligible remedy known to us. Or in other words, by a judicious use of mercury, at the right moment, the recovery may be materially accelerated, and thus organic and functional lesions may be avoided. I suppose that good practice consists not alone in conducting our patient successfully through their sickness, but taking them by the shortest route, and the medicine or means that do just that, is the most eligible. I believe it to be entirely demonstrable that mercury has been and can be made to control certain affections whose ultimate limit without it would be a matter of months instead of weeks. Still other cases that without it would last week after week, that by its use may be cured in as many days. And it seems to me that no amount of ingenuity of argument deduced from its excessive to common or indiscreet use, ought for a moment to prejudice us against it.

Dr. Jansen remarked that he agreed with Dr. Rochester in regard to the treatment of iritis. Have treated a great many cases of syphilitic iritis and always with mercury. Have seen three cases treated without it with total loss of sight in every case.

Dr. White remarked that he did not arise to take part in the discussion or to dissent from the conclusions arrived at but to correct an error that *may* be sent abroad by what has been said in regard to the practice of the profession in Buffalo. The battle against the too liberal and indiscriminate use of mercury was fought and won twenty years ago. With all my experience at home and abroad, I make the broad assertion that there is less calomel given in Buffalo than in any of the other cities. Twenty years ago we carried the matter too far and gave too little. In our attempts at reform we must not go too far.

Dr. Gay remarked that he did not feel like permitting the discussion to close without giving expression to his appreciation of the value of the paper read by Dr. Lothrop. Under the first heading the doctor has introduced the American theory of the action of mercury without, as I understand, advocating the theory itself. The remnrks of Dr. Rochester upon this branch of the subject are timely and weighty, and although I could add further proof of the falacy of the theory, I will not occupy the time necessary.

Would say a word in reference to the other topic touched upon in the paper and in the discussion, viz: the use of mercury as an aplastic. For convenience medicine might be divided into two periods of twenty years each, more or less—the mercurial and the anti-mercurial periods. Dr. White has passed through the former and is far advanced in the latter period, and has given his testimony to the great change wrought in the administration of mercury during this period of time. We may justly infer from his remarks that the day is past for the administration of mercury for its aplastic properties. I had long labored under the conviction that the action of mercury upon a serous membrane when given largely to children, and no person will doubt that during the mercurial period mercury was given to children to excess, because of the difficulty of causing ptyalism, and also the action of mercury upon serous membranes when given to adults to the point of salivation was deleterious, and would impart to those serous structures a lesion which would be felt in after life. If it be not a popular error, it is at least a popular belief that sometimes acute and chronic arthritis are in some way chargeable to mercurialization. Should there be any measure of truth in this popular belief that mercury has been an agent of distruction or injury to membranous coverings of the joints, who is able to estimate the amount of structural change produced directly by the same agency in the membranous covering of the heart, or indirectly the heart itself? Again should there be any measure of truth in such popular belief, then be assured that the injuries inflicted in childhood and manifested in adult life are but the ingathering of the harvest springing up from seed sown during the mercurial period.

Election of officers being the next order of business, an election was held with the following result:

For President, SANDFORD EASTMAN, M. D.
" Vice President, J. R. LOTHROP, M. D.
" Secretary, T. M. JOHNSON, M. D.
" Treasurer, T. T. LOCKWOOD, M. D.
" Librarian, J. B. SAMO, M. D.

By vote of the Association the President and Secretary were directed to give the proper credentials to four delegates to represent this Association at the next meeting of the American Medical Association.

Adjourned. T. M. JOHNSON, *Secretary.*

